# Health-Related Quality of Life in Spanish Schoolchildren and Its Association with the Fitness Status and Adherence to the Mediterranean Diet

**DOI:** 10.3390/nu14112322

**Published:** 2022-05-31

**Authors:** Rosario Pastor, Cristina Bouzas, Irene Albertos, Carolina García, Ángela García, Teresa Prieto, Jorge Velázquez, Elena Sánchez-Jiménez, Rocío Rodríguez, Francisco Javier Martín, Angélica María Campón, Josep A. Tur

**Affiliations:** 1 Research Group on Community Nutrition and Oxidative Stress, University of the Balearic Islands-IUNICS, 07122 Palma de Mallorca, Spain; rosario.pastor@ucavila.es (R.P.); cristina.bouzas@uib.es (C.B.); 2 Faculty of Health Sciences, Catholic University of Avila, 05005 Avila, Spain; rene.albertos@ucavila.es (I.A.); carolina.garcia@ucavila.es (C.G.); angela.garcia@ucavila.es (Á.G.); teresa.prieto@ucavila.es (T.P.); jorge.velazquezsaornil@ucavila.es (J.V.); elena.sanchez@ucavila.es (E.S.-J.); rocio.rodriguez@ucavila.es (R.R.); fjavier.martin@ucavila.es (F.J.M.); angelicacampon@gmail.com (A.M.C.); 3 CIBEROBN (Physiopathology of Obesity and Nutrition), Instituto de Salud Carlos III, 28029 Madrid, Spain; 4 Foundation Health Research Institute of the Balearic Islands (IDISBA), 07120 Palma de Mallorca, Spain

**Keywords:** health-related quality of life, fitness, Mediterranean diet, schoolchildren

## Abstract

Background: Health-related quality of life (HRQoL) allows knowing the subject’s feelings in distress and well-being, as well as perception of current and future health. Objective: To assess associations between health-related quality of life, fitness status, and adherence to the Mediterranean diet in Spanish children and adolescents. Methods: A cross-sectional study was carried out in a sample of 305 (47.2% women) children and adolescents aged between 8 and 16 years, in the primary and secondary schools of the province of Ávila (central Spain) (NCT05380674). Subjects were classified according to their quality of life: group 1 (highest quality of life) > group 2 (medium quality of life) > group 3 (lowest quality of life). Results: More participants in group 1 showed higher adherence to Mediterranean diet (70.8%) than other groups (group 2: 55.0%; group 3: 43.4%). It was less likely to find optimal levels of muscle strength as quality of life decreased (OR; 95% CI: group 2: 0.535; 0.303–0.955; and group 3: 0.424; 0.234–0.768). Similar trends were found for speed and agility, but only group 3 showed significant results (OR; 95% CI: group 3: 0.297; 0.162–0.545). Flexibility was also the worst in groups 2 and 3 (OR; 95% CI: G2: 0.403; 0.213–0.762; and group 3: 0.520; 0.282–0.958). Conclusion: High fitness status and adherence to the Mediterranean diet were associated with improved health-related quality of life in schoolchildren of central Spain.

## 1. Introduction

Health-related quality of life (HRQoL) is an expression of the subject’s well-being that encompasses the individual’s social, mental, and physical dimensions [1]. Therefore, it allows knowing the subject’s feelings in terms of distress and well-being, as well as their perception of their current and future health [2]. It has been established that functional status, well-being, and general health are the basic components of HRQoL, which are essential to be able to meet the demands of daily lives, as well as to satisfy people’s needs and desires [3]. Self-reported HRQoL losses are a measure of general population health, allowing comparisons between different groups; it is increasingly considered in economic evaluations [4,5].

People’s quality of life is influenced by multiple factors that can affect them throughout their lives. Some models have determined that HRQoL can be affected by demographic aspects such as age, gender, or education, by issues related to current health status, and by environmental factors [2,6,7]. Nutritional status, given its importance for the appropriate functions of human beings, should be closely related to HRQoL. However, scientific evidence did not yet support it, although a trend has been observed relating a worse quality of life in subjects with a deteriorated nutritional status, especially when populations with pathologies were studied. Likewise, grip strength is associated with quality of life. Low grip strength values are indicative of muscle dysfunction and are directly related to poorer quality of life [8,9].

Healthy eating habits during adolescence play an important role in overall well-being, including health-related quality of life, and can positively affect the development of psychological well-being and brain maturation [10,11]. There is also evidence that adherence to the Mediterranean diet pattern is positively associated with adolescent well-being and mental health [12,13].

In young populations, most of the research has evaluated the quality of life of youths with some pathology. It has been observed that HRQoL changes according to the disease and its severity, as well as the treatment received, which can improve the quality of life of patients [14,15,16,17]. In Spanish healthy young people, without illness, a very low prevalence of severe problems has been observed, as might be expected, with the most affected dimensions being “feeling worried, sad or unhappy” and “having pain or discomfort” [18].

Therefore, few studies have studied the association between health-related quality of life with physical state and adherence to the Mediterranean diet. To address this research need, the aim of the current study was to assess the associations between health-related quality of life, fitness status, and adherence to the Mediterranean diet in Spanish children and adolescents.

## 2. Materials and Methods

### 2.1. Design and Subjects

**Figure 1:**
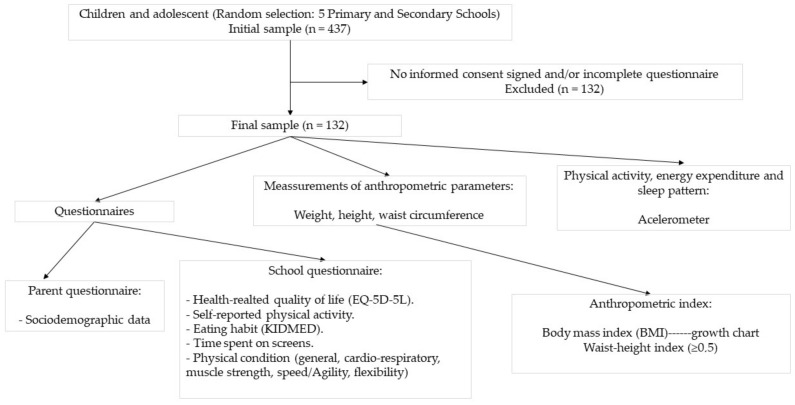
Flowchart of the study.

A cross-sectional study was carried out in an initial sample of 437 children and adolescents aged between 8 and 16 years, in the primary and secondary schools of the province of Ávila (Spain), during the academic year 2020–2021. Participants were randomly selected in five schools, four public and one concerted, located in different areas of the province, where families of medium socioeconomic status reside. The following exclusion criteria were applied: serious illness, which may influence the nutritional or functional status of the child, and mental limitations of the parents and/or children, which may hinder the completion of the questionnaire. From the initial sample selected, 132 children did not present the informed consent signed by the parents or legal guardians and/or submitted an incomplete questionnaire (parent questionnaire or school questionnaire) and were excluded from the study. The final sample consisted of 305 schoolchildren (47.2% women). Figure 1 shows the study protocol.

The study protocol followed the Declaration of Helsinki ethical standards, and all procedures were approved by the Ethics Committee of the Ávila Province (ref. GASAV 2020/13). All parents were informed of the purpose of the study and informed consent was obtained by each participating child, which was signed by children and parents. The trial was registered in the Clinical Trial registry (https://clinicaltrials.gov/) with the number NCT05380674, (accessed on 27 May 2022).

### 2.2. Questionnaires

#### 2.2.1. Parent Questionnaire, Study Information Sheet, and Informed Consent

The short questionnaire of healthy habits for adults of the PASOS (Physical Activity, Sedentarism and Obesity in Spanish Youth) study was used [19], which also collects sociodemographic data, among others, which are not relevant for the purpose of the present paper. Parents and/or legal guardians of the children completed the questionnaire either online or on paper, according to the preferred route chosen by the directors of the centers.

The information sheet about the study and the informed consent were distributed in the classrooms by the teachers, to be signed by the parents and/or legal guardians of the children.

#### 2.2.2. School Questionnaire

The questionnaire of healthy habits of children and adolescents of the PASOS (Physical Activity, Sedentarism and Obesity in Spanish Youth) study was used [19], which collects the following data:

- Health-related quality of life: Questions from the EQ-5D-5L questionnaire (EuroQol-5 Dimensions-5 levels), which will be discussed in depth later;
- Self-reported physical activity habits;
- Eating habits: Questionnaire of 16 quality questions of the Mediterranean diet in childhood and adolescence on which the KIDMED index [20] is based, which we will also delve into later;
- Time spent on screens;
- Hours of sleep and quality of rest;
- Questions about physical condition (general, cardio-respiratory, muscle strength, speed/agility, flexibility).

### 2.3. Meassurement of Anthropometric Parameters

Weight, height, and waist circumference were measured following standardized protocols of the World Health Organization [21]. To measure the weight, an electronic digital scale (model TANITA BC-545N) (weight capacity: 150 kg; accuracy: 0.1 kg) was used. A telescopic mechanical stadiometer (SECA 222, SECA Deutschland, Hamburg, Germany; measuring range: 6–230 cm; accuracy: 1 mm) was used to measure the height. To measure the waist circumference, a flexible and non-elastic tape was used (SECA 201, SECA Deutschland, Hamburg, Germany; measuring range: 0–205 cm; width ≤7 mm; accuracy: 1 mm; with an ungraded space before zero and with an easy-to-read scale). All measurements were performed three times and the arithmetic mean of the three values was used for analysis. Subjects were classified according to the body mass index (BMI) sex and age specific cut-off points previously established in the growth chart of Fernández, et al. [22]. A waist-to-height index (IQ) ≥ 0.5 was used as indicator of abdominal fat accumulation and increased health risk in children and adolescents [23].

### 2.4. Adherence to the Mediterranean Diet

To evaluate the adherence to the Mediterranean diet of schoolchildren, the quality index of the Mediterranean diet for children and adolescents (KIDMED), validated for the Spanish child and youth population in the EnKid study [20], was used. This index can vary between 0 and 12 and is based on a questionnaire of 16 questions that can be answered in the affirmative or negative. This questionnaire is included in the global questionnaire completed by the schoolchildren, described above. Affirmative answers to questions that reflect a positive aspect related to the Mediterranean diet add one point and affirmative answers to questions that represent a negative aspect in relation to the Mediterranean diet subtract one point. The sum of the scores obtained allows to classify the subjects in three levels of adherence to the Mediterranean diet: ≥8 or optimal Mediterranean diet; 4–7 or need for improvements in the eating pattern to adapt it to the Mediterranean model; ≤3 or very low-quality diet.

### 2.5. Physical Activity, Energy Expenditure, and Sleep Pattern

Participants wore an accelerometer (ActiGraph wGT3X-B; ActiGraph LLC, Pensacola, FL, USA) on the dominant wrist, with supervision from parents and researchers for proper placement. Detailed instructions were provided to both participating children and parents to facilitate compliance. They were instructed to use the accelerometer for 7 consecutive days, both during waking and sleeping hours, and not to remove it for bathing or water activities, unless these were performed at a depth greater than 1 m and/or for more than 30 min.

To evaluate physical activity, energy expenditure due to physical activity and sleep pattern, the accelerometer records obtained in the sample of school children were interpreted. To establish the energy expenditure by physical activity (kcal/day) and the levels of physical activity (sedentary, light, moderate to vigorous), from the data collected in the accelerometer, the equation and the cut-off points established by Freedson, et al. [24,25] were used. Moreover, the Cole–Kripke sleep algorithm was used to obtain sleep efficiency (minutes/day, %/day).

### 2.6. Health-Related Quality of Life

To assess the quality of life of schoolchildren, the EuroQol-5 Dimensions-5 levels (EQ-5D-5L) validated questionnaire was used. The EuroQol Group developed the EQ-5D-5L as a generic health status questionnaire that assesses the quality of daily life [26]. This questionnaire has been validated both for the healthy population and for the population with pathologies [27,28] and makes it possible to quantitatively express the situation in which the subject finds themself referring to five dimensions of their health status [29,30]. This tool makes it possible to detect variations in HRQoL as well as clinical changes that different subjects may experience [31].

The EQ-5D-5L instrument contains five dimensions: mobility, self-care, daily activities, pain/discomfort, and concerns/sadness. Each dimension, in turn, contains five levels: no problems, mild problems, moderate problems, severe problems, and extreme problems. Thus, 5^5^ (i.e., 3125) possible health states can be described. Each health problem can be described using a five-digit code, so that the code 11,111 describes the best state of health, and the code 55555 indicates the worst possible state of health. The instrument also includes a visual analogue scale (VAS) on general health: “We would like to know how good or bad your health is TODAY (list from 0 to 100, with 100 being the best health you can imagine and 0 being the worst health you can imagine)” [32].

The EuroQol group designed a standardized protocol to assess health status from the EQ-5D-5L questionnaire, using the EQ-5D-5L index, trying to minimize heterogeneity by creating a set of EQ-5D-5L index values for different countries. [33]. Following this protocol and using the set of values for the Spanish population, an EQ-5D-5L index was obtained for the sample of schoolchildren in our study, with a range of 1 (perfect health) to negative values (for those health states considered worse than death).

The grouping variable was “Salud HOY/100 + Eq-5D-5l index value”. The first was divided by 100 so that the two had the same weight in the results. To obtain the tertile classification, a grouping variable was calculated by the sum of the variables “SaludHOY/100” + “EQ5D5Lindexvalue”. As a result, three groups with a similar number of subjects were obtained. The cut-off percentiles were: Group 1: >1.932; Group 2: 1.932–1.782; Group 3: <1.782.

To analyze the variable “Health of TODAY” (EVA), the subjects were classified according to the following Overall Health Category Rating Scales (CRS): Poor (<53); Fair (≥53 and <76); Good (≥76 and <80); Very Good (≥80 and <90); Excellent (≥90).

From the values assigned to the five dimensions of the EQ-5D-5L, a severity index was calculated as follows: Severity Index (SI) = (ΣValues assigned to the 5D − 5) × 5. The values of this index range from 0 to 100 (SI = 0, total absence of problems; SI = 100, higher degree of severity). Subsequently, this IS was transformed into a health index (HI), with the aim of being able to make a comparison with the EQ-5D-5L index, since in the latter, the highest values correspond to better health states (HI = 100 − SI).

### 2.7. Statistics

Statistical analyses were performed using IBM SPSS^®^ Statistics software version 28.0 (SPSS Inc., Chicago, IL, USA). Subjects were categorized into three groups according to tertiles of the life quality variable. The quantitative variables were expressed as the mean ± standard deviation (SD). The qualitative variables were analyzed with measures of frequency and percentages. Difference in means between groups were tested by one-way ANOVA and Bonferroni’s post-hoc. Differences in prevalence’s across groups were calculated by means of χ^2^. A linear regression was carried out between adherence to the Mediterranean diet (MD) and life quality (dependent variable). Odds Ratios (OR) were calculated for the physical state of children variables. All tests were considered statistically significant if *p*-value (two-tailed) < 0.05.

## 3. Results

### 3.1. Sociodemographic Characteristics of Parent and Children and Answers to the EQ-5D-5L According to Health-Related Quality of Life

Table 1 shows sociodemographic characteristics of parents and children according to health-related quality of life. Children reporting a lower life quality were older than children reporting higher levels of life quality.

Table 2 shows answers to the EQ-5D-5L. Participants with higher life quality (Group 1) reported higher health scores, as well as better answers to each of the questions on the EQ-5D-5L. Group 3 reported the lowest health scores and less optimal answers to the EQ-5D-5L. As health decreased, so did health-related quality of life. All participants in Group 1 had excellent health according to category rating scales (CRS), while only 61% and 11% of Groups 2 and 3, respectively, were classified into that category.

### 3.2. Health Parameters and Health-Related Behaviours, according to Health-Related Quality of Life

Health parameters and health-related behaviors are available in Table 3. No differences among groups were found for energy expenditure, physical activity, sleeping pattern, BMI categorization, or abdominal obesity prevalence. However, more participants in Group 1 had higher adherences to Mediterranean diet (70.8%), compared to the other groups (Group 2: 55.0%; Group 3: 43.4%). More participants in Group 3 reported medium (47.5%) and low adherence (9.1%) than in the other groups (medium adherence: Group 2: 42.0%, Group 1: 26.4%; low adherence: G2: 3.0%, Group 1: 2.8%).

**Figure 2:**
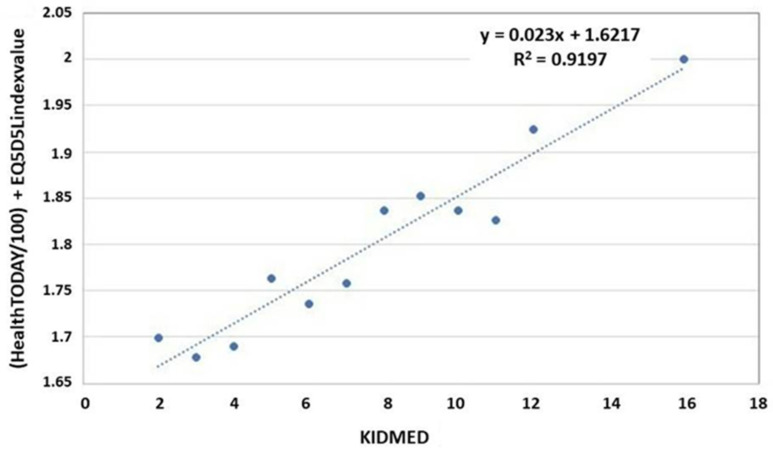
Correlation between health-related quality of life and adherence to the Mediterranean diet.

A higher adherence to Mediterranean diet was found among participants reporting a higher life quality. Such findings were further explored, as shown in Figure 2, which shows a positive correlation between life quality and Mediterranean diet adherence. As Mediterranean adherence increases, so does health-related quality of life.

### 3.3. Fitness Status of Children according to Life Quality

Table 4 shows the fitness status of children according to health-related quality of life. As well as the health of the day in which interviews were conducted did not seem to have any relationship with reported quality of life. However, no participant in the best quality of life group (Group 1) was experiencing any pain/discomfort or concerns/sadness, while higher percentages of participants experienced pain/discomfort or concerns/sadness in the other groups, with Group 3 being the one with the worst levels of pain/discomfort and concerns/sadness (Pain/discomfort: some: Group 2: 6.0%, Group 3: 14.1%; a lot: Group 2: 2.0%, Group 3: 2.0%. Concerns/sadness: some: Group 2: 15.0%, Group 3: 24.2%; a lot: Group 2: 1.0%, Group 3: 1.0%). Participants reporting higher levels of life quality had better levels of muscle strength, speed and agility, and flexibility.

### 3.4. Association between the Fitness Status of Children and Their Health-Related Quality of Life

Table 5 shows the association between the fitness status of children and their health-related quality of life. It was less likely to find optimal levels of muscle strength as quality of life decreased (OR (95% CI): Group 2: 0.535 (0.303–0.955); Group 3: 0.424 (0.234–0.768). Same tendency was found for speed and agility; however, only Group 3 had an OR statistically significant (OR (95% CI): G2: 0.584 (0.334–1.021); Group 3: 0.297 (0.162–0.545). Flexibility was also worst in Groups 2 and 3 (OR (95% CI): G2: 0.403 (0.213–0.762); G3: 0.520 (0.282–0.958). Hence, a lower reported quality of life was associated with a less optimal fitness status.

## 4. Discussion

In the current study, it was observed that the quality of life of young people was related to the fitness status of the subjects. These results agree with those obtained in previous research [34,35], which showed a positive association between physical fitness, cardiorespiratory fitness, and muscular fitness with quality of life in a sample of Portuguese adolescents. In the United States population aged 9–11 years, a relationship between quality of life and cardiorespiratory fitness and muscular fitness was also reported, although no such relationship was observed with flexibility [36]. In the current study, a relationship seems to be observed between flexibility and quality of life, since significant differences were observed among groups as well as in the quality of life. The fitness status of young people is closely related to the practice of physical activity; studies carried out in the population in different Spanish regions showed that physical activity had a positive relationship with quality of life, being greater when the amount of sports practice carried out by adolescents is higher [37,38,39].

Furthermore, situations in which a change in the patterns of physical activity carried out by young people has been observed, such as the COVID-19 lockdown or following a training program, are not only related to changes in fitness status but also to variations in health-related quality of life, especially referred to “Physical Wellbeing” [40,41]. Therefore, we can point out that physical activity is a fundamental component of health-related quality of life and that a sedentary lifestyle not only results in poorer health in the population but also in a reduction in their quality of life.

A connection between nourishment and the quality of life of adolescents seems to also observed. Research carried out in Chile [42] and Portugal [34] showed the importance of a healthy eating pattern and specifically with adherence to the Mediterranean Diet on the quality of life in young people. This issue was confirmed by a previous study carried out on Spanish population [38], in which the Mediterranean diet pattern was related to the psychological well-being and better perception of the school environment, resulting in better quality of life of adolescents; it was attributed to adherence to Mediterranean diet regardless of sex or BMI. Likewise, in a sample of Spanish adolescents, an association of adherence to the Mediterranean and happiness levels was observed, through specific components of health-related quality of life [12]. These results agree with those of the current study, in which it was observed that subjects with better perceived health and quality of life had greater adherence to the Mediterranean diet pattern.

The current research did not show a relationship between the BMI of young people and the self-perceived health-related quality of life. These results are consistent with those observed in the Chinese child population, in which no significant relationship was observed between children’s BMI and their health-related quality of life [43]. However, previous studies analyzing the health-related quality of life in Spanish adolescents showed a relation between it and BMI, showing that overweight and obese adolescents had worse quality of life score [37]. Another study on Chilean adolescents showed an inverse relationship between BMI and quality of life [42].

This controversy may be because, as previously stated [44], the influence of BMI on health-related quality of life is less than that observed by other factors, such as physical activity. These authors observed that BMI was only significantly associated with the Physical Wellbeing and Psychological Wellbeing domains of health-related quality of life. Likewise, it was observed that the relationship between BMI and health-related quality of life was more pronounced in the case of females, corroborating the complexity between both variables and the multitude of aspects that can influence it [45]. The current research demonstrated that both physical activity and adherence to the Mediterranean diet, both closely related to BMI, were associated with health-related quality of life, and it may be that these factors are the ones that are really interacting in our sample.

The current research also found no relationship between physical activity levels and health-related quality of life. The association between physical activity and health-related quality of life in children and adolescents was primarily assessed among those with chronic diseases, such as obesity, cancer, or asthma [46,47,48,49]. These studies concluded that children and adolescents who led an active lifestyle had a better quality of life than those who were sedentary. Regarding the population without pathologies, the relationship between health-related quality of life and physical activity has been well established [50], but much less is known about this relationship in children and adolescents. A systematic review [51] pointed out that high levels of physical activity were associated with higher health-related quality of life, whereas sedentary lifestyle was inversely related. However, even though longitudinal studies and some intervention studies included in this review demonstrated that the physical activity of children and adolescents influenced future health-related quality of life, a causal effect cannot be concluded since the number of trials is still too small.

Regarding sleep patterns, it has been observed in female adolescents that sleep quality interacts with menstrual health and together they affect the quality of life [52]. In fact, the presence of sleep-related pathologies, such as obstructive sleep apnea, is directly related to health-related quality of life in young people [53]. Despite this, in our research, no such relationship was found with the sleeping pattern, perhaps because the groups contained subjects of both sexes.

### Strengths and Limitations

The current study provides information on health-related quality of life and its association with fitness status and adherence to the Mediterranean diet in Spanish adolescents, a topic of worldwide relevance. The evaluation of health-related quality of life in children and adolescents is important to identify poor health states and to design intervention strategies to improve their health. Although the current study advances our understanding of the relationship between health-related quality of life with fitness and adherence to the Mediterranean diet in children and adolescents, it has certain limitations. First, this is a cross-sectional study, so evidence of association can be established, but not causality; thus, more longitudinal studies are needed. Furthermore, the sample is not representative of all Spanish children and adolescents, but only of central Spanish children and adolescents. This is a limitation to the generalizability of the results to the general Spanish population. Another limitation is that health-related quality of life, fitness, and adherence to the Mediterranean diet have been assessed using self-reported questionnaires; its potential for bias is well known, although quality control was followed to minimize this bias.

Finally, it is important to highlight that the data in our study were collected during the COVID-19 pandemic. Thus, the results may be affected, since during this stage, children have undergone changes that have affected their lifestyle, as evidenced by different existing publications in the literature [54,55,56,57,58,59,60].

## 5. Conclusions

High fitness status and adherence to the Mediterranean diet were associated with improved health-related quality of life in schoolchildren of central Spain.

Further longitudinal research would be needed to assess causality. Research including other southern European regions is advisable to extrapolate results to the general population. The current results highlight the need to develop health programs aimed at establishing healthy lifestyles in children and adolescents, which could be useful to design health promotion programs.

## Figures and Tables

**Table 1:** Sociodemographic characteristics of parents and children according to health-related quality of life.

	Group 1 ^§^ (*n* = 106)	Group 2 ^§^ (*n* = 100)	Group 3 ^§^ (*n* = 99)	*p*-Value ^‡^
	Mean (SD)	Mean (SD)	Mean (SD)	
Parent age (years)	44.65 (4.7)	44.77 (5.5)	43.81 (7.0)	>0.05
Parent BMI (kg/m^2^)	25.56 (4.4)	24.35 (4.7)	25.25 (4.0)	>0.05
Child age	10.42 (1.7)	10.47 (1.4)	11.04 (1.9)	0.016
	*n* (%)	*n* (%)	*n* (%)	
Child gender (female)	54 (50.9)	49 (49.0)	41 (41.4)	>0.05
Parent education level				>0.05
Illiterate	2 (1.9)	2 (2.0)	0 (0.0)	
Primary school	14 (13.2)	7 (7.0)	4 (4.0)	
Middle school	10 (9.4)	6 (6.0)	12 (12.1)	
Vocational training	19 (17.9)	18 (18.0)	17 (17.2)	
High school	9 (8.5)	13 (13.0)	15 (15.2)	
University studies	38 (35.9)	35 (35.0)	27 (27.3)	
Not reported	14 (13.2)	19 (19.0)	24 (24.3)	
Parent employment status				>0.05
Homemaker	7 (6.6)	4 (4.0)	4 (4.0)	
Working	77 (72.7)	70 (70.0)	59 (59.6)	
Unemployed	7 (6.6)	4 (4.0)	8 (8.1)	
Others	1 (0.9)	3 (3.0)	4 (4.0)	
Not reported	14 (13.2)	19 (19.0)	24 (24.2)	
Parent smoking habit				>0.05
Current smoker	17 (16.0)	11 (11.0)	20 (20.2)	
Former smoker (0–1 year)	2 (1.9)	0 (0.0)	3 (3.0)	
Former smoker (1–5 years)	5 (4.7)	3 (3.0)	6 (6.1)	
Former smoker (>5 years)	17 (16.0)	16 (16.0)	13 (13.1)	
Never smoked	51 (48.2)	52 (52.0)	32 (32.3)	
Not reported	14 (13.2)	18 (18.0)	25 (25.3)	

Abbreviations: BMI: Body Mass Index; SD: Standard deviation. ^§^ Grouping variable = ((HealthTODAY/100) + EQ5D5Lindexvalue). The cut-off percentiles were as follows: Group 1: >1.932; Group 2: 1.932–1.782; Group 3: <1.782. ^‡^ Difference in means between groups were tested by one-way ANOVA and Bonferroni’s post-hoc. Differences in prevalence’s across groups were examined using χ^2^.

**Table 2:** Answers of the EQ-5D-5L of children according to health-related quality of life.

	Group 1 ^§^ (*n* = 106)	Group 2 ^§^ (*n* = 100)	Group 3 ^§^ (*n* = 99)	*p*-Value ^‡^
	Mean (SD)	Mean (SD)	Mean (SD)	
Health Index (HI)	98.3 (2.1) ^a,b^	88.9 (5.8) ^a,c^	69.2 (16.0) ^b,c^	<0.001
Domains of the questionnaire	*n* (%)	*n* (%)	*n* (%)	
1. Mobility (walking) TODAY				<0.001
I can’t/Very bad	0 (0.0)	0 (0.0)	0 (0.0)	
Many problems/Bad	0 (0.0)	0 (0.0)	1 (1.0)	
Quite a few problems/Acceptable	0 (0.0)	0 (0.0)	1 (1.0)	
Any problem/Good	0 (0.0)	2 (2.0)	20 (20.2)	
No problems/Very good	106 (100.0)	98 (98.0)	77 (77.8)	
2. Self-care TODAY				0.001
I can’t/Very bad	0 (0.0)	0 (0.0)	0 (0.0)	
Many problems/Bad	0 (0.0)	0 (0.0)	1 (1.0)	
Quite a few problems/Acceptable	0 (0.0)	0 (0.0)	1 (1.0)	
Any problem/Good	0 (0.0)	2 (2.0)	11 (11.1)	
No problems/Very good	106 (100.0)	98 (98.0)	86 (86.9)	
3. Daily activities TODAY				<0.001
I can’t/Very bad	0 (0.0)	0 (0.0)	0 (0.0)	
Many problems/Bad	0 (0.0)	0 (0.0)	2 (2.0)	
Quite a few problems/Acceptable	0 (0.0)	0 (0.0)	0 (0.0)	
Any problem/Good	0 (0.0)	7 (7.0)	23 (23.3)	
No problems/Very good	106 (100.0)	93 (93.0)	74 (74.7)	
4. Pain/discomfort or feeling bad TODAY				<0.001
I can’t/Very bad	0 (0.0)	0 (0.0)	2 (2.0)	
Many problems/Bad	0 (0.0)	0 (0.0)	2 (2.0)	
Quite a few problems/Acceptable	0 (0.0)	1 (1.0)	3 (3.0)	
Any problem/Good	0 (0.0)	12 (12.0)	26 (26.3)	
No problems/Very good	106 (100.0)	87 (87.0)	66 (66.7)	
5. Concerns/sadness TODAY				<0.001
I can’t/Very bad	0 (0.0)	0 (0.0)	3 (3.0)	
Many problems/Bad	0 (0.0)	0 (0.0)	3 (3.0)	
Quite a few problems/Acceptable	0 (0.0)	4 (4.0)	4 (4.0)	
Any problem/Good	8 (7.5)	18 (18.0)	22 (22.3)	
No problems/Very good	98 (92.5)	78 (78.0)	67 (67.7)	
6. Overall health category rating scales (CRS)				<0.001
Poor (<53)	0 (0.0)	0 (0.0)	22 (22.2)	
Fair (≥53 y <76)	0 (0.0)	0 (0.0)	47 (47.5)	
Good (≥76 y <80)	0 (0.0)	1 (1.0)	1 (1.0)	
Very Good (≥80 y <90)	0 (0.0)	38 (38.0)	18 (18.2)	
Excellent (≥90)	106 (100.0)	61 (61.0)	11 (11.1)	

Abbreviations: EQ-5D-5L, Euroqol-5 Dimensions-5 levels; SD, Standard deviation. ^§^ Grouping variable = ((HealthTODAY/100) + EQ5D5Lindexvalue). The cut-off percentiles were as follows: Group 1: >1.932; Group 2: 1.932–1.782; Group 3: <1.782. ^‡^ Difference in means between groups were tested by one-way ANOVA and Bonferroni’s post-hoc. Differences in prevalence’s across groups were examined using χ^2^. * Different letters indicate statistically significant differences (*p* < 0.05) between groups * tertiles (^a,b,c^) by the Bonferroni post-hoc test.

**Table 3:** Health parameters and health-related behaviors in children according to health-related quality of life.

	Group 1 ^§^ (*n*= 106)	Group 2 ^§^ (*n* = 100)	Group 3 ^§^ (*n* = 99)	*p*-Value ^‡^
	Mean (SD)	Mean (SD)	Mean (SD)	
Energy expenditure (kcal/day) *	568.9 (332.9)	491.8 (97.25)	866 (558.9)	>0.05
Physical activity (minute/day) *				
Sedentary	474.2 (183.7)	563.4 (73.7)	588.5 (33.4)	>0.05
Light	424.8 (149)	476.2 (50.2)	499.5 (56.8)	>0.05
Moderate to vigorous	256.5 (78.9)	265.8 (52.8)	257.3 (73.4)	>0.05
Sleeping pattern *				
Sleep efficiency (%/day)	92.3 (3.0)	91 (4.0)	92.7 (3.2)	>0.05
Sleeping time (min/day)	399.1 (99.6)	408.6 (102.9)	400.7 (33.1)	>0.05
	*n* (%)	*n* (%)	*n* (%)	
BMI percentile *				>0.05
Underweight	6 (7.7)	8 (11.4)	5 (8.2)	
Normal weight	55 (70.5)	48 (68.6)	40 (65.6)	
Overweight	12 (15.4)	8 (11.4)	12 (19.7)	
Obesity	5 (6.4)	6 (8.6)	4 (6.5)	
Abdominal obesity (yes) *	11 (55)	12 (50)	4 (22)	>0.05
MedDiet adherence				0.001
Low adherence	3 (2.8)	3 (3.0)	9 (9.1)	
Medium adherence	28 (26.4)	42 (42.0)	47 (47.5)	
High adherence	75 (70.8)	55 (55.0)	43 (43.4)	

Abbreviations: BMI, Body Mass Index; MedDiet, Mediterranean Diet; SD, Standard deviation. ^§^ Grouping variable = ((HealthTODAY/100) + EQ5D5Lindexvalue). The cut-off percentiles were: Group 1: >1.932; Group 2: 1.932–1.782; Group 3: <1.782. ^‡^ Difference in means between groups were tested by one-way ANOVA and Bonferroni’s post-hoc. Differences in prevalence’s across groups were examined using χ^2^.

**Table 4:** Fitness status of children according to health-related quality of life.

	Group 1 ^§^ (*n* = 106)	Group 2 ^§^ (*n* = 100)	Group 3 ^§^ (*n* = 99)	*p*-Value ^‡^
	*n* (%)	*n* (%)	*n* (%)	
Muscle strength				0.001
Very bad	0 (0)	0 (0)	2 (2.0)	
Bad	4 (3.8)	2 (2.0)	5 (5.1)	
Acceptable	10 (9.4)	19 (19.0)	31 (31.3)	
Good	45 (42.5)	49 (49.0)	36 (36.4)	
Very good	47 (44.3)	30 (30.0)	25 (25.2)	
Speed and agility				0.005
Very bad	1 (0.9)	0 (0)	2 (2.0)	
Bad	3 (2.8)	2 (2.0)	8 (8.1)	
Acceptable	10 (9.4)	13 (13.0)	17 (17.2)	
Good	40 (37.7)	49 (49.0)	50 (50.5)	
Very good	52 (49.0)	36 (36.0)	22 (22.2)	
Flexibility				0.004
Very bad	3 (2.8)	4 (4.0)	6 (6.1)	
Bad	7 (6.6)	11 (11.0)	12 (12.1)	
Acceptable	26 (24.5)	27 (27.0)	35 (35.4)	
Good	31 (29.3)	39 (39.0)	23 (23.2)	
Very good	39 (36.8)	19 (19.0)	23 (23.2)	
Pain/discomfort				0.010
None	106 (100)	92 (92.0)	83 (83.8)	
Some	0 (0)	6 (6.0)	14 (14.2)	
A lot	0 (0)	2 (2.0)	2 (2.0)	
Concerns/sadness				0.010
None	106 (100)	84 (84.0)	74 (74.8)	
Some	0 (0)	15 (15.0)	24 (24.2)	
A lot	0 (0)	1 (1.0)	1 (1.0)	

Abbreviations: SD, Standard deviation. ^§^ Grouping variable = ((HealthTODAY/100) + EQ5D5Lindexvalue). The cut-off percentiles were: Group 1: >1.932; Group 2: 1.932–1.782; Group 3: <1.782. **^‡^** Difference in means between groups were tested by one-way ANOVA and Bonferroni’s post-hoc. Differences in prevalence’s across groups were examined using χ^2^.

**Table 5:** Association between the fitness status of children and their life quality.

	Group 1 ^§^ (*n* =106)	Group 2 ^§^ (*n* = 100)	Group 3 ^§^ (*n* = 99)
	OR (95% CI)	OR (95% CI) *p*-Value	OR (95% CI) *p*-Value
Muscle strength	1.00 (ref.)	0.535 (0.303–0.955) 0.034	0.424 (0.234–0.768) 0.005
Speed and Agility	1.00 (ref.)	0.584 (0.334–1.021) 0.059	0.297 (0.162–0.545) < 0.001
Flexibility	1.00 (ref.)	0.403 (0.213–0.762) 0.005	0.520 (0.282–0.958) 0.036
Pain/discomfort	1.00 (ref.)	1.506 (0.866–2.620) 0.147	1.065 (0.616–1.843) 0.821
Concerns/sadness	1.00 (ref.)	1.053 (0.608–1.825) 0.853	0.916 (0.529–1.586) 0.753

Abbreviations: OR, Odds Ratio. CI, Confidence Interval. ^§^ Grouping variable = ((HealthTODAY/100) + EQ5D5Lindexvalue). The cut-off percentiles were: Group 1: >1.932; Group 2: 1.932–1.782; Group 3: <1.782.

## Data Availability

There are restrictions on the availability of data for this trial, due to the signed consent agreements around data sharing, which only allow access to external researchers for studies following the project purposes. Requestors wishing to access the trial data used in this study can make a request to pep.tur@uib.es.
